# Identifying the Challenges of Child Abuse Detection Among Emergency, Pediatrics, and Family Medicine Practitioners in Saudi Arabia

**DOI:** 10.7759/cureus.38022

**Published:** 2023-04-23

**Authors:** Naif M AlShalhoub, Abdulaziz A Bin shalhoub, Hussam E Alshanawani, Saleh A Showail, Shams A Alowais, Zahi M Alhamad, Bandar S Almutairi, Sultan T Alturki, Abdulrahman Al-mana

**Affiliations:** 1 Family Medicine, Prince Sultan Military Medical City, Riyadh, SAU; 2 Forensic Medicine, Forensic Medicine Center, Riyadh, SAU; 3 Forensic Medicine, Saudi Ministry of Health (MOH), Riyadh, SAU; 4 Forensic Medicine, Ministry of Health (MOH), Riyadh, SAU; 5 Medicine, Imam Mohammad Ibn Saud Islamic University, Riyadh, SAU; 6 General Practice, Prince Sultan Military Medical City, Riyadh, SAU; 7 Emergency Medicine, Prince Sultan Military Medical City, Riyadh, SAU; 8 Family and Community Medicine, Prince Sultan Military Medical City, Riyadh, SAU

**Keywords:** child abuse in emergency department, child trauma, child abuse, neglect, abuse

## Abstract

Background: Child abuse is a significant issue across many countries. Despite the situation's innate understanding, many children are not reported to authorities and continue to experience abuse, sometimes even death. Healthcare professionals must be alert for abuse in any child who appears with injuries that are out of the ordinary because it is easy for indicators of child abuse to go unnoticed in a busy emergency department. The current study aims to evaluate and detect the challenges in diagnosing and reporting cases of child abuse among healthcare practitioners in emergency, pediatrics, and family medicine.

Methods: A self-administered online disseminated questionnaire was used for data collection during the period from October 1 to December 30, 2022. A cross-sectional study was conducted on emergency, pediatrics, and family medicine healthcare practitioners working in hospitals in healthcare centers in Riyadh, Saudi Arabia. All data were collected, tabulated, and statistically analyzed using SPSS 23.0 for (IBM Corp., Armonk, NY) Windows.

Results: The study sample constituted 200 physicians working in the front lines of healthcare like emergency, pediatrics, and family medicine primary care services, 50.5% were males and 49.5% were females. 36.5% of participants were 31-39 years old. 42% were family medicine physicians, 36.5% were pediatricians, and 21.5% were emergency medicine. About 43% of participants attended an educational workshop on child abuse. Nineteen percent of participants are very familiar with the diagnosis of child abuse and 36% of participants reported one to three cases of child abuse in the emergency department in the last year, 5% reported four to six cases and 56.5% reported none. Forty-seven percent of participants reported diagnosing one to five cases of child abuse throughout their whole career, 13% reported 11-15 cases, 6.5% reported six to 10 cases and 28.5% reported none. Causes of underdiagnosis of child abuse by healthcare providers were reported as 63% inexperience, 59% inadequate time for physical examination, 59% lack of diagnosis protocol, 51% lack of confidence in communicating with parents, 36% physicians' cultural background, and 38% lack of confidence in the diagnosis. 93.5% of participants think that healthcare practices need further education for child abuse.

Conclusion: In conclusion, physicians in Saudi Arabia who participated in the study had good knowledge to diagnose a case of child abuse. Inexperience, inadequate time for physical examination, lack of diagnosis protocol, lack of confidence in communicating with parents, and physicians' cultural background were the main identified challenges for diagnosing child abuse. Familiarity with cases of child abuse was significantly associated with physicians’ age, specialty, and level of training.

## Introduction

Child abuse is a public health concern that has long-term effects on both physical and mental health [1]. The World Health Organization estimates that 40 million children worldwide experience sexual abuse every year, while 23% also experience physical abuse and 36% experience emotional abuse [2]. The likelihood of physical child abuse decreased dramatically (by 47.7%) during the COVID-19 epidemic. The COVID-19 pandemic had no statistically significant impact on children's chances of experiencing emotional abuse, according to one study [3].

Abuse-related head trauma can result in physical disabilities such as cerebral palsy, developmental delays, and neurologic abnormalities. Psychologically, those who have suffered from child abuse are more likely to experience depression, conduct disorders, and substance dependence. These kids may perform poorly academically and have impaired cognitive abilities [4].

Despite having the desire to increase the detection of child abuse, health professionals do not have the time to create suitable policies and protocols, register (suspicions of) child abuse, or plan education and training [5]. Additionally, the large patient number in the ED and the wide range of symptom intensity make it challenging for clinicians to calmly discuss a suspect of child abuse with parents [6].

A thorough assessment of the various types and severity of child abuse in Saudi Arabia was conducted in 2018 by reading publications written on the subject during the previous 25 years as a result of this. It was discovered that child abuse is regrettably still an issue in Saudi Arabia, and its prevalence is of concern to both the government and the healthcare agencies. Incidents are reported much less frequently than they actually occur, typically because the victim was innocent or because investigating agencies were callous and insensitive [3,7]. A meta-analysis of 15 research on child maltreatment conducted in Saudi Arabia in 2019 revealed that the prevalence of child abuse is high, with physical abuse and neglect, in particular, being the most commonly reported types of abuse throughout the 15 studies, with an overall prevalence rate of 15% [8].

All healthcare professionals have a moral, legal, and professional duty to recognize child abuse when they see it and to report it to authorities. Since the Emergency Department sees the majority of child abuse cases, nurses and doctors are frequently the first to identify the issue. The first step is to recognize the issue; allowing abused children to go back to their abusers frequently results in increased violence, and occasionally even fatalities. The social worker must be notified even if there is only a remote possibility that child abuse occurred so that the child can be monitored as an outpatient [9,10]. The current study aims to explore what practicing physicians in Primary Care Services (like an emergency, pediatrics, and family medicine) perceive as the challenges to diagnosing and reporting cases of child abuse.

## Materials and methods

Study type and duration

A cross-sectional study was conducted during the period from October 1 to December 30, 2022 on physicians in front lines specialties in some of Riyadh hospitals and Healthcare centers, who were invited to participate and fill out the questionnaire of the study. Research clearance and approval were obtained from the Ethical Research Committee of the Medical Services Department for Armed Forces Scientific Research Center.

Sample size

The sample size was 200 emergency, pediatrics, and family medicine healthcare practitioners working in hospitals in healthcare centers in Riyadh, Saudi Arabia, in order to achieve sound results. The calculation methodology of sample size, Raosoft, was used for the population survey.

Data collection tool and technique

A pre-designed online questionnaire was uploaded on Google Services and was used for data collection. The questionnaire link was sent to the target population via WhatsApp platform. The questionnaire was adopted from multiple studies, which addressed the subject of healthcare practitioners dealing with child abuse and its determinants. It was mainly composed of two main sections as followed:

Section 1 included sociodemographic characteristics of the participants, e.g., age, sex, marital status, nationality, occupational characteristics, degree, and years of experience. Section 2 asked about experiences and attitudes toward child abuse and barriers to reporting it. The Likert scale was used for some questions. Question regarding the previous diagnosis of a case of child abuse, frequency of evaluating such cases, and settings of diagnosis.

The survey was filled out by the participants personally. The questionnaire had a brief introduction explaining the nature of the research and highlighting the anonymity of the participants.

Ethical considerations

The ethical issue in the study states that avoiding asking the participants about certain races or genders of the patients or asking the participants to admit any mistake that he or one of his colleagues made. Research clearance and approval were obtained from the Ethical Research Committee of the Medical Services Department for Armed Forces Scientific Research Center. This work has been carried out in accordance with The Code of Ethics of the World Medical Association (Declaration of Helsinki) for studies involving humans.

Data management and analysis

All data were collected, tabulated, and statistically analyzed using SPSS 23.0 (IBM Corp., Armonk, NY) for Windows. Qualitative data were stated as absolute frequency and relative frequency (percentage). Proportions of categorical variables were compared using either the chi-square test or Fisher's exact test. P-value < 0.05 was considered statistically significant.

## Results

As illustrated in Table 1, the study included 200 participants, 50.5% were males and 49.5% were females. 36.5% of participants were 31-39 years old, 33.5% were 30 years or less and 30% were 40 years or more. 48.5% were married with children, 16.5% were married without children and 35% were single. As for specialty, 42% were family medicine, 36.5% were pediatricians and 21.5% were emergency medicine. 35.5% of participants were consultants and 19% were specialists. 36.5% work in military hospitals, 22% work in general Ministry of Health (MOH) hospitals, and 19.5% in private hospitals. 90.5% of participants live in urban areas, 6.5% in suburban areas, and 3.5% in rural areas.

**Table 1:** Sociodemographic characteristics of participants (n=200)

Parameter		No.	%
Age	30 years old or less	67	33.5
	31-39 years old	73	36.5
	40 years old or more	60	30.0
Gender	Male	101	50.5
	Female	99	49.5
Marital status	Married with children	97	48.5
	Married without children	33	16.5
	Single	70	35.0
Speciality	Emergency Medicine	43	21.5
	Family medicine	84	42.0
	Paediatrics	73	36.5
Level of medical training	R1	15	7.5
	R2	32	16.0
	R3	30	15.0
	R4	10	5.0
	Consultant	71	35.5
	Resident	4	2.0
	Specialist (Registrar or Senior registrar)	38	19.0
Type of institution	Military hospital	73	36.5
	MOH hospital	44	22.0
	Private hospital	39	19.5
	Security forces hospital	4	2.0
	Specialized hospital	12	6.0
	Teaching hospital (University hospital)	28	14.0
Current residence location	Rural	7	3.5
	Suburban	12	6.0
	Urban	181	90.5
Experience as medical practitioner	1 to 5 years	72	36.0
	6 to 10 years	55	27.5
	more than 10 years	73	36.5

Table 2 illustrates participants’ experience with child abuse incidents. 43% of participants attended an educational workshop on child abuse and 84.5% are interested in attending one. 19% of participants are very familiar with the diagnosis of child abuse and 21.5% estimated the highest level of confidence in diagnosing a case of child abuse. 36% of participants reported one to three cases of child abuse in the emergency department in the last year, 5% reported four to six cases and 56.5% reported none. 47% of participants reported diagnosing one to five cases of child abuse throughout their whole career, 13% reported 11-15 cases, 6.5% reported six to 10 cases and 28.5% reported none.

**Table 2:** Participants’ experience with child abuse (n=200)

Parameter		No.	%
Attended an educational workshop on child abuse	Yes	86	43.0
	No	114	57.0
Interested in attending one in the future	Yes	169	84.5
	No	26	13.0
	I don't know	5	2.5
Familiar with diagnosis of child abuse	1	11	5.5
	2	20	10.0
	3	62	31.0
	4	69	34.5
	5	38	19.0
Estimate level of confidence in diagnosing cases of child abuse	1	9	4.5
	2	19	9.5
	3	68	34.0
	4	61	30.5
	5	43	21.5
Cases of child abuse diagnosed in 2021	1-3	70	36.0
	4- 6	10	5.0
	More than 6	7	3.5
	None	113	56.5
Cases of child abuse diagnosed through career	0	57	28.5
	5-1	94	47.0
	10-6	13	6.5
	15-11	26	13.0
	15+	10	5.0
Can detect child abuse as early as possible	Yes	147	73.5
	No	53	26.5

Regarding attitude, Table 3 shows that 6% strongly agree that abuse is only physical, 10% strongly agree that abused children grow up to be abusers, 20% strongly agree that they can identify abused children, 38.5% strongly agree that abusers have an unstable social life, 53% strongly agree that disabled children are likely to become a victim of abuse and 7.5% strongly agree that practitioners generally receive adequate training in child abuse.

**Table 3:** Participants’ attitude towards child abuse (n=200)

Parameter		No.	%
Its only abuse if it was physical	1	114	57.0
	2	24	12.0
	3	17	8.5
	4	33	16.5
	5	12	6.0
Abused children always grow up to be abusers	1	15	7.5
	2	43	21.5
	3	71	35.5
	4	51	25.5
	5	20	10.0
I can identify children who are abused	1	6	3.0
	2	25	12.5
	3	63	31.5
	4	66	33.0
	5	40	20.0
Child abusers tends to have an unstable social life	1	11	5.5
	2	13	6.5
	3	27	13.5
	4	72	36.0
	5	77	38.5
Disabled children are likely to become victims of abuse	1	4	2.0
	2	11	5.5
	3	28	14.0
	4	51	25.5
	5	106	53.0
Practitioners generally receive adequate training in child abuse	1	32	16.0
	2	64	32.0
	3	56	28.0
	4	33	16.5
	5	15	7.5
Practitioners generally receive adequate training in child abuse	1	34	17.0
	2	59	29.5
	3	62	31.0
	4	32	16.0
	5	13	6.5
Practitioners generally receive adequate training in child abuse	1	31	15.5
	2	59	29.5
	3	57	28.5
	4	38	19.0
	5	15	7.5

Table 4 shows that causes of underdiagnosis of child abuse by healthcare providers were reported as 63% inexperience, 59% inadequate time for physical examination, 59% lack of diagnosis protocol, 51% lack of confidence in communicating with parents, 36% physicians' cultural background and 38% lack of confidence in the diagnosis. 93.5% of participants think that healthcare practices need further education for child abuse. 47% are familiar with medical laws regulating child abuse in Saudi Arabia. 89% recommend adding courses on child abuse to public schools. 86.5% recommend improving child abuse detection in practice by improving child abuse training during residency, 64% recommend offering child abuse CME courses and 51% recommend developing subspecialty training in child abuse.

**Table 4:** Causes of underdiagnosis of child abuse by healthcare providers

Parameter		No.	%
Cause of underdiagnosis of child abuse by healthcare providers (Bias may occur)	Physicians cultural background	72	36.0
	Lack of confidence in communicating with parents	102	51.0
	Lack of confidence in diagnosis	76	38.0
	Inexperience	126	63.0
	Lack of diagnosis protocol	118	59.0
	Inadequate training	44	22.0
	Inadequate time for physical examination	118	59.0
	language barrier	26	13.0
	Inadequate training	114	57.0
Healthcare practices need further education for child abuse	Yes	187	93.5
	No	13	6.5
Familiar with medical laws regulating child abuse in Saudi Arabia	Yes	94	47.0
	No	101	50.5
	I don't know	5	2.5
Recommend adding courses on child abuse to public school	Yes	178	89.0
	No	17	8.5
	I don't know	5	2.5
Recommend to improve child abuse detection in practice	Improve child abuse training during residency	173	86.5
	Offer child abuse CME course	128	64
	Develop a subspecialty training in child abuse	102	51
	None	2	1

Table 5 shows that familiarity with diagnosing child abuse was significantly associated with the age and level of medical training of participants (P=0.001).

**Table 5:** Association between sociodemographic characteristics of participants with familiarity with diagnosing child abuse

		Familiar with diagnosis of child abuse		Total (N=200)	P value
		No	Yes		
Age	30 years old or less	48	19	67	0.001
		51.6%	17.8%	33.5%	
	31-39 years old	28	45	73	
		30.1%	42.1%	36.5%	
	40 years old or more	17	43	60	
		18.3%	40.2%	30.0%	
Gender	Male	51	50	101	0.253
		54.8%	46.7%	50.5%	
	Female	42	57	99	
		45.2%	53.3%	49.5%	
Marital status	Married with children	40	57	97	0.158
		43.0%	53.3%	48.5%	
	Married without children	14	19	33	
		15.1%	17.8%	16.5%	
	Single	39	31	70	
		41.9%	29.0%	35.0%	
Specialty	Emergency Medicine	17	26	43	0.037
		18.3%	24.3%	21.5%	
	Family medicine	48	36	84	
		51.6%	33.6%	42.0%	
	Pediatrics	28	45	73	
		30.1%	42.1%	36.5%	
Level of medical training	R1	12	3	15	0.001
		12.9%	2.8%	7.5%	
	R2	23	9	32	
		24.7%	8.4%	16.0%	
	R3	16	14	30	
		17.2%	13.1%	15.0%	
	R4	4	6	10	
		4.3%	5.6%	5.0%	
	Consultant	19	52	71	
		20.4%	48.6%	35.5%	
	Resident	2	2	4	
		2.2%	1.9%	2.0%	
	Specialist (Registrar or Senior registrar)	17	21	38	
		18.3%	19.6%	19.0%	
Type of institution	Military hospital	36	37	73	0.855
		38.7%	34.6%	36.5%	
	MOH hospital	20	24	44	
		21.5%	22.4%	22.0%	
	Private hospital	15	24	39	
		16.1%	22.4%	19.5%	
	Security forces hospital	2	2	4	
		2.2%	1.9%	2.0%	
	Specialised hospital	5	7	12	
		5.4%	6.5%	6.0%	
	Teaching hospital (University hospital)	15	13	28	
		16.1%	12.1%	14.0%	
Current residence location	Rural	4	3	7	0.815
		4.3%	2.8%	3.5%	
	Suburban	6	6	12	
		6.5%	5.6%	6.0%	
	Urban	83	98	181	
		89.2%	91.6%	90.5%	

## Discussion

When dealing with the problem of child abuse and neglect, front-line physicians in emergency, pediatrics, and family medicine have a crucial role to play and face particular difficulties such as inadequate training [3]. This study aimed to assess awareness of child abuse among practitioners Working on the front lines and providing a primary care service and to identify the challenges in diagnosing and reporting child abuse cases.

According to our study results, 19% of participants are very familiar with diagnosing child abuse, and 21.5% estimated the highest level of competency in diagnosing a case of child abuse. 36% of participants reported one to three cases of child abuse in the emergency department in the last year, 5% reported four to six cases and 56.5% reported none. Forty-seven percent of participants reported diagnosing one to five cases of child abuse throughout their whole career, 13% reported 11-15 cases, 6.5% reported six to 10 cases and 28.5% reported none. This was consistent with earlier research, according to Kraus and Jandl-Jager's report that the majority of doctors were aware of the most typical signs of child abuse [11]. According to Alnasser et al., Saudi medical students, pediatric residents, and pediatricians possess solid foundational knowledge [12]. Another study by Habib found that participants generally had a good understanding of some key elements of child abuse and neglect [13]. Li et al. stated that there was a lack of awareness of child maltreatment among health professionals in China [14]. This distinction can result from the study's inclusion of all health professionals. Yadav and Datta noted that most family doctors lacked adequate information regarding the recognition and treatment of child abuse cases [15]. Additional studies, like Hynniewta et al.'s assessment of awareness among other crucial individuals like teachers, are necessary for the detection and prevention of child abuse and neglect [16]. 84.5% of participants stated a wish to attend an educational program on child abuse, but only 43% did, raising the possibility that there may be a lack of training as a contributing factor. Twenty percent of Saudis who participated in a different study reported having heard a lecture or attended a session on child abuse and neglect within the previous five years or more [17].

In our study, causes of underdiagnosing child abuse by healthcare providers were reported as 63% inexperience, 59% inadequate time for physical examination, 59% lack of diagnosis protocol, 51% lack of confidence in communicating with parents, 36% physicians' cultural background and 38% lack of confidence in the diagnosis. A Saudi study conducted in Jeddah found that barriers prevent reporting cases of suspected child abuse because 78.6% of respondents were unsure if the kid had been molested [17]. This can suggest that the information and abilities needed to recognize the signs and symptoms of abuse are lacking. These findings are in agreement with those of Alrimawi et al., Ragan and Olympio, Lynne and Gifford, and Skarsaune and Bondas [18-21]. The failure to recognize abuse signs and symptoms is an obvious obstacle to reporting child maltreatment, according to Alvarez et al., [22]. Lack of knowledge about reporting procedures and/or prior negative experiences with reporting that had an impact on the family or the child are additional obstacles that prevent reporting child abuse and neglect [17]. In addition, Lynne et al. [20] discovered that 38% of respondents were uninformed about the reporting mechanism for child abuse. The findings of a study on the role of healthcare professionals' knowledge as one of the factors influencing an individual's decision to report showed that more pediatricians with limited knowledge and the inability to recognize children with genital abnormalities declined to report cases of suspected child abuse [7]. But there are situations when ignorance is not the primary excuse for failing to report suspected cases. Because of this, some professionals may be hesitant to disclose suspected cases of child abuse even when their expertise grows [9]. Merrild and Frost found that the ambiguous sign of child abuse made the detection and reporting challenging, hence they felt that they lack chances to report such cases [23]. Foster et al. either found that a big percentage of healthcare practitioners rarely screen for child maltreatment as they do feel uncomfortable in discussing such matters, as additional training is needed to improve healthcare system outcomes [24].

According to our study results, most participants think that healthcare practices need further education regarding child abuse. A cross-sectional study in Nigeria reported being unfamiliar with policies regarding the diagnosis and reporting of child abuse and advised increasing training for medical professionals on child abuse to be able to assist such patients [25]. According to a cross-sectional study by Starling et al., the resident's understanding and approach to such situations need to be improved by the residency programs [26].

In our study, familiarity with diagnosing a case of child abuse was significantly associated with physicians’ age and level of training. According to Aldukhayel et al. [27], primary healthcare physicians in Al-Khobar City, Eastern Region of Saudi Arabia, had a general knowledge of child abuse that was significantly higher among those between the ages of 36 and 40. This finding is consistent with our findings regarding the age of the respondents. They also claimed that having more experience was related to having more knowledge [27].

Programs for training doctors on child abuse should evaluate their prior knowledge, target their interventions to a particular group of doctors, and specify their behavioral and educational goals [28]. It is important to consider the individual needs of students while developing educational programs for medical professionals and students. Physicians with different specializations and degrees of training are likely to have distinct educational demands because there are variances in knowledge and comfort levels across the many medical professions [9]. On this basis, 89% of our study participants recommend adding courses on child abuse to public schools. 86.5% recommend improving child abuse detection in practice by improving child abuse training during residency, 64% recommend offering child abuse courses and 51% recommend developing subspecialty training in child abuse.

When authorities create training programs to inform other health professionals about child abuse, they should take all of the cited impediments into account [13]. The current study's findings show that simply inviting all medical professionals to workshops and lectures on child abuse and neglect is insufficient because many nurses simply cannot find time in their hectic schedules for such training.

## Conclusions

In conclusion, physicians in Riyadh seem to have acceptable knowledge to diagnose a case of child abuse compared to previously published literature worldwide. Lack of experience, inadequate time for physical examination, lack of diagnosis protocol, lack of confidence in communicating with parents, and physicians' cultural background were the main identified challenges for diagnosing child abuse. Familiarity with cases of child abuse was significantly associated with physicians’ age, specialty, and level of training. Children must be guarded, and any suspected cases of child abuse must be reported and documented to the appropriate authorities so they can look into them and aid the children. It is necessary to conduct an adequate inquiry and take measures to support abuse victims in order to alleviate the harm that is caused to these children and their families. Another way to raise awareness and knowledge about reporting child abuse is through additional training, which should be considered necessary.

## Appendices

Questionnaire: Identifying the challenges of child abuse detection among Emergency, Pediatrics, and Family medicine practitioners in Saudi Arabia

We are a group of medical practitioners working in different hospitals across the Kingdom of Saudi Arabia conducting this research to identify the challenges and difficulty of child abuse detection among health care practitioners in the Emergency, pediatrics, and Family medicine departments.

Our goals from this research:

1-   To identify the challenges in diagnosing and in reporting such cases.

2-   To assess awareness of child abuse among practitioners in different specialities.

Estimated time to answer the questionnaire: 3-5 minutes

      * Indicates required question                                      

1.         Age group *

Mark only one oval.

30 years old or less 31-39 years old

40 years old or more

2.         Gender *

Mark only one oval.

Male Female

3.         Marital status *

Mark only one oval.

Single

Married with children Married without children

4.         What is your speciality? *

Mark only one oval.

Emergency Medicine Pediatrics

Family medicine

5.         What is your level of medical training? *

Mark only one oval.

R1 R2 R3 R4

Specialist ( Registrar or Senior registrar)

Consultant

Other:                                      

6.         Which of the following best describes   *

your institution?

Mark only one oval.

Teaching hospital (University hospital)

Military hospital MOH hospital Private hospital Specialised hospital

Other:                                      

7.         Which of the following best describes   *

your current location of practice?

Mark only one oval.

Urban Rural Suburban

8.         How long have you worked as a            *

medical practitioner?

Mark only one oval.

1 to 5 years

6 to 10 years

more than 10 years

9.         Have you ever attended an                   *

educational workshop on child abuse?

Mark only one oval.

Yes No

10.         Would you be interested in attending *

one in the future?

Mark only one oval.

Yes No

11.         How familiar are you with the               *

diagnosis of child abuse

Mark only one oval.

Not familiar

1

2

3

4

5

very familiar

12.         Estimate your level of competence in *

diagnosing cases of child abuse

Mark only one oval.

significantly below average

1

2

3

4

5

significantly above average

13.         How many cases of child abuse did     *

you diagnose/report in 2021?

Mark only one oval.

None 1-3

4-6

More than 6

14.         Throughout your career, how many                                                   * cases of child abuse have you reported total? (estimate)

Mark only one oval.

0

1-5

5-10

10-15

20+

15.         Do you believe you can detect child    *

abuse as early as possible?

Mark only one oval.

Yes No

16.         Estimate your level of agreement with the following statements:

Check all that apply.

Ok

17.         "It's only abuse if it was physical" *

Mark only one oval.

strongly disagree

1

2

3

4

5

strongly agree

18.         "Abused children always grow up to    *

be abusers"

Mark only one oval.

strongly disagree

1

2

3

4

5

strongly agree

19.         "I can identify children who are            *

abused"

Mark only one oval.

strongly disagree

1

2

3

4

5

strongly agree

20.         "Child abusers tends to have an          *

unstable social life"

Mark only one oval.

strongly disagree

1

2

3

4

5

strongly agree

21.         "Disabled children are likely to             *

become victims of abuse"

Mark only one oval.

strongly disagree

1

2

3

4

5

strongly agree

22.         "Practitioners generally receive                                                * adequate training in child abuse evaluation"

Mark only one oval.

strongly disagree

1

2

3

4

5

strongly agree

23.         "Practitioners generally receive                                                * adequate training in child abuse diagnosis"

Mark only one oval.

strongly disagree

1

2

3

4

5

strongly agree

24.         "Practitioners generally receive                                                * adequate training in child abuse management"

Mark only one oval.

strongly disagree

1

2

3

4

5

strongly agree

25.         What do you think is the cause of the * under diagnosis of child abuse by healthcare providers?

Check all that apply.

Inexperience

Lack of con\dence

Lack of diagnosis protocol Lack of con\dence in

communicating with parents

Inadequate training Inadequate time for physical

examination

language barrier

physicians cultural background

Other:                                       

26.         Do you think that health care               *

practitioners need further education on child abuse?

Mark only one oval.

Yes No

27.         Are you familiar with medical laws       *

regarding child abuse in Saudi Arabia

Mark only one oval.

Yes No

28.         Were you taught in primary schooling * (elementary-high school) about child abuse?

Mark only one oval.

Yes No

29.         Would you recommend adding                                                 * courses on child abuse to public school curriculums?

Mark only one oval.

Yes No

30.         What would you recommend to                                                         * improve child abuse detection in your practice

Check all that apply.

Improve child abuse training during residency

Offer child abuse CME course Develop a subspecialty training in

child abuse

None

Other:                                       

This content is neither created nor endorsed by Google.

Forms
